# Targeted deletion of *Crif1* in mouse epidermis impairs skin homeostasis and hair morphogenesis

**DOI:** 10.1038/srep44828

**Published:** 2017-03-20

**Authors:** Jung-Min Shin, Dae-Kyoung Choi, Kyung-Cheol Sohn, Ji-Young Kim, Myung Im, Young Lee, Young-Joon Seo, Minho Shong, Jeung-Hoon Lee, Chang Deok Kim

**Affiliations:** 1 Departmentof Dermatology, Chungnam National University School of Medicine, Daejeon, Korea; 2 Division of Endocrinology, Department of Internal Medicine, Research Center for Endocrine and Metabolic Diseases, Chungnam National University School of Medicine, Daejeon, Korea

## Abstract

The epidermis, which consists mainly of keratinocytes, acts as a physical barrier to infections by regulating keratinocyte proliferation and differentiation. Hair follicles undergo continuous cycling to produce new one. Therefore, optimum supply of energy from the mitochondria is essential for maintaining skin homeostasis and hair growth. CRIF1 is a mitochondrial protein that regulates mitoribosome-mediated synthesis and insertion of mitochondrial oxidative phosphorylation polypeptides into the mitochondrial membrane in mammals. Recent studies reveal that conditional knockout (cKO) of *Crif1* in specific tissues of mice induced mitochondrial dysfunction. To determine whether the mitochondrial function of keratinocytes affects skin homeostasis and hair morphogenesis, we generated epidermis-specific *Crif1* cKO mice. Deletion of *Crif1* in epidermis resulted in impaired mitochondrial function and *Crif1* cKO mice died within a week. Keratinocyte proliferation and differentiation were markedly inhibited in *Crif1* cKO mice. Furthermore, hair follicle morphogenesis of *Crif1 c*KO mice was disrupted by down-regulation of Wnt/β-catenin signaling. These results demonstrate that mitochondrial function in keratinocytes is essential for maintaining epidermal homeostasis and hair follicle morphogenesis.

The epidermis is a self-renewing multilayered epithelium that forms a barrier against external insults as well as excessive fluid loss. Epidermal homeostasis is continuously regulated by both proliferation and differentiation of keratinocytes. Hair follicles, present beneath the epidermis, cycle through three phases, namely, anagen (growing phase), catagen (regression phase) and telogen (resting phase). Since the development and differentiation are affected by mitochondrial function in many biological systems^1^,^2^,^3^,^4^, it is plausible that epidermal events including keratinocyte differentiation and hair cycling are regarded as the processes to which a large quantity of energy is applied to.

Mitochondria provide many cellular functions, such as energy production, fatty acid/amino acid oxidation, iron metabolism and apoptosis^5^. The major function of mitochondria is to generate ATP through oxidative phosphorylation (OXPHOS). Mitochondrial ribosomes encode the genetic information supplied by the mitochondrial DNA (mtDNA) to synthesize 13 OXPHOS proteins at the inner mitochondrial membrane^6^.

CRIF1 was first identified as a CR6/GADD45-interacting protein which negatively regulates cell growth and apoptosis^7^. As a nuclear protein, CRIF1 acts as co-activator of transcription factors such as STAT3 and Elf3^8^,^9^,^10^,^11^. However, a recent study shows that CRIF1 is a mitochondrial protein which regulates the synthesis and insertion of OXPHOS polypeptides by interacting with the mitoribosomal large subunit^12^. Previous studies have reported that conditional knockout (cKO) mice of *Crif1* in specific tissues, such as brain, heart, intestine and adipose tissues, induced mitochondrial dysfunction^13^,^14^,^15^,^16^.

Involvement of mitochondrial metabolism in epidermal differentiation and hair growth has been reported. Conditional knockout of *Tfam*, which regulates mitochondrial transcription and replication, impairs epidermal differentiation and hair development^17^. Hormones involved in the hypothalamic-pituitary-thyroid (HPT) axis regulate hair growth by stimulating mitochondrial gene expression and mitochondrial biogenesis within human hair follicles^18^. Furthermore, the relationship between mitochondrial dysfunction and skin diseases has been documented^19^. Despite the importance of mitochondrial function in skin biology, evidence supporting the notion is limited.

In this study, we generated epidermis-specific *Crif1* cKO mice. These *Crif1* cKO mice showed mitochondrial dysfunction, reduced keratinocyte differentiation, and disturbed hair follicle morphogenesis. Our data provide evidence that *Crif1* is essential for epidermal homeostasis and hair follicle morphogenesis and exerts its effects by regulating mitochondrial function.

## Results

### Deletion of *Crif1* in mouse epidermis

**Figure 1:**
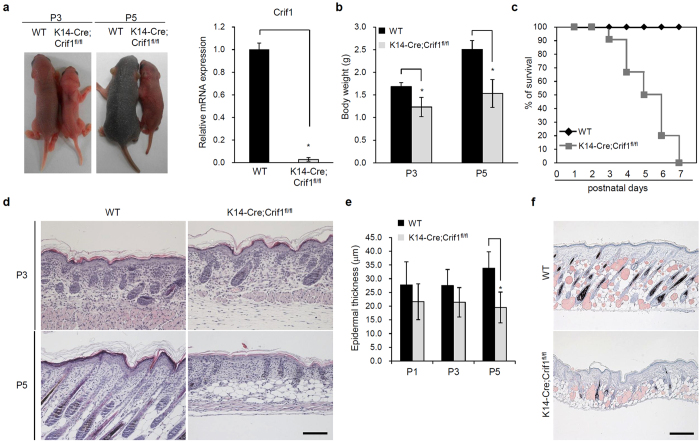
Targeted deletion of *Crif1* in mouse epidermis. (**a**) Images comparing body size and hair growth of WT and *Crif1* cKO mice at P3 and P5 (left). qPCR analysis of *Crif1* mRNA levels in epidermal lysates from WT and *Crif1* cKO mice (P5) (right). (**b**) Analysis of body weight of WT and *Crif1* cKO mice demonstrating reduced body size of *Crif1* cKO mice (n = 5 mice per group). (**c**) Survival analysis of WT and *Crif1*cKO mice (n = 15 mice per group). (**d**) Histological examination of the epidermis using hematoxylin and eosin (H&E)-stained skin sections from WT and *Crif1* cKO mice at P3 and P5. Scale bar, 100 µm. (**e**) Quantification of epidermal thickness of WT and *Crif1* cKO mice (n = 5 mice per group). (**f**) Oil Red O staining of subcutaneous fat layer and sebaceous glands in WT and *Crif1* cKO at P5. Scale bar, 50 μm. *P < 0.01.

We generated the *Crif1* cKO mice by crossing *K14-Cre* and *Crif1*^*fl/fl*^ mice. In all the experiment, knockout pups (*K14-Cre;Crif1*^*fl/fl*^) were compared with wild type (WT) littermates (*Crif1*^*fl/fl*^). Targeted deletion of *Crif1* in the epidermis was verified by quantitative-PCR and Western blot analysis. The *Crif1*cKO pups showed growth retardation and abnormal appearance (Figs 1a, S1). Compared to wild type (WT) mice, *Crif1* cKO pups showed slower body weight gain (Fig. 1b) and died within a week (Fig. 1c). Histological analysis of the dorsal skin of *Crif1* cKO mice at postnatal day 3 (P3) revealed normal phenotype with slightly reduced epidermal thickness compared to that of the WT mice. Hair follicle morphogenesis appeared to be similar to that of WT mice. However, at P5, significant differences were observed between the cKO and WT mice; hair follicle morphogenesis was severely hampered, there was shrinkage of pre-established hair follicles (Fig. 1d), and epidermal thickness was significantly reduced (Fig. 1e). Oil Red O staining revealed that the subcutaneous fat layer and mature sebaceous glands were remarkably decreased in *Crif1* cKO mice (Fig. 1f). Morphology of other epithelial tissues of the *Crif1* cKO mice, such as esophagus and intestine, was similar to that of WT mice (Figure S2). Heterozygous (*K14-Cre; Crif1*^*fl*/+^) mice were normal in phenotype and had no survival defect (Figure S3).

### Mitochondrial dysfunction following loss of *Crif1*

**Figure 2:**
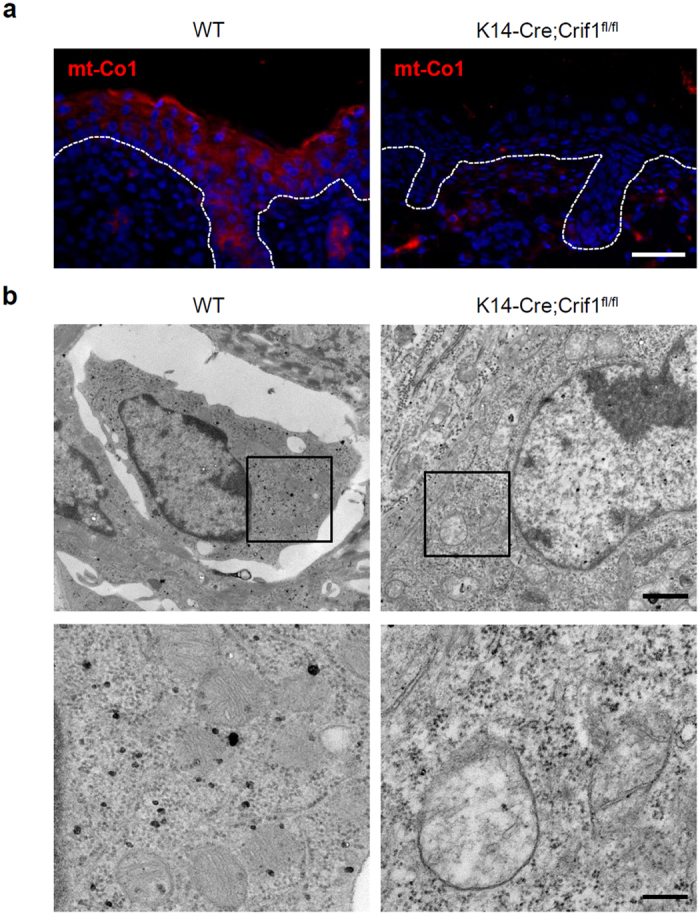
Mitochondrial dysfunction following *Crif1* loss. (**a**) Immunofluorescence staining of mt-Co1 in skin sections of WT and *Crif1* cKO mice (P5) (red, mt-Co1; blue, DAPI). Scale bar, 200 μm. (**b**) Transmission electron microscopic images of the epidermis of WT and *Crif1* cKO mice (P5). Abnormal mitochondria were examined in *Crif1* cKO epidermis. Black squares indicate the enlarged regions. Scale bar, 1 μm (upper panel), 200 nm (lower panel).

Mitoribosomes translate the genetic information supplied by mtDNA to synthesize 13 OXPHOS proteins at the inner mitochondrial membrane. CRIF1 controls the synthesis and insertion of OXPHOS polypeptides by interacting with the large mitoribosomal subunit. Ablation of *Crif1* leads to aberrant synthesis and defective insertion of mtDNA-encoded nascent OXPHOS polypeptides into the inner membrane^12^. To determine whether mitochondrial dysfunction occurs in *Crif1*-deleted epidermis, we investigated the expression of mt-Co1 (an mtDNA-encoded subunit of OXPHOS) by immunohistochemistry. The expression of mt-Co1 was significantly reduced in the epidermis of *Crif1* cKO mice (Figs 2a, S4). Transmission electron microscopic images of mitochondria in the epidermis of *Crif1* cKO showed morphological abnormalities, including a loss of electron dense material from the matrix and distorted or reduced numbers of cristae (Fig. 2b). These results indicated that *Crif1* was essential for the integrity of mitochondrial structure.

### Impaired keratinocyte differentiation in *Crif1* cKO

**Figure 3:**
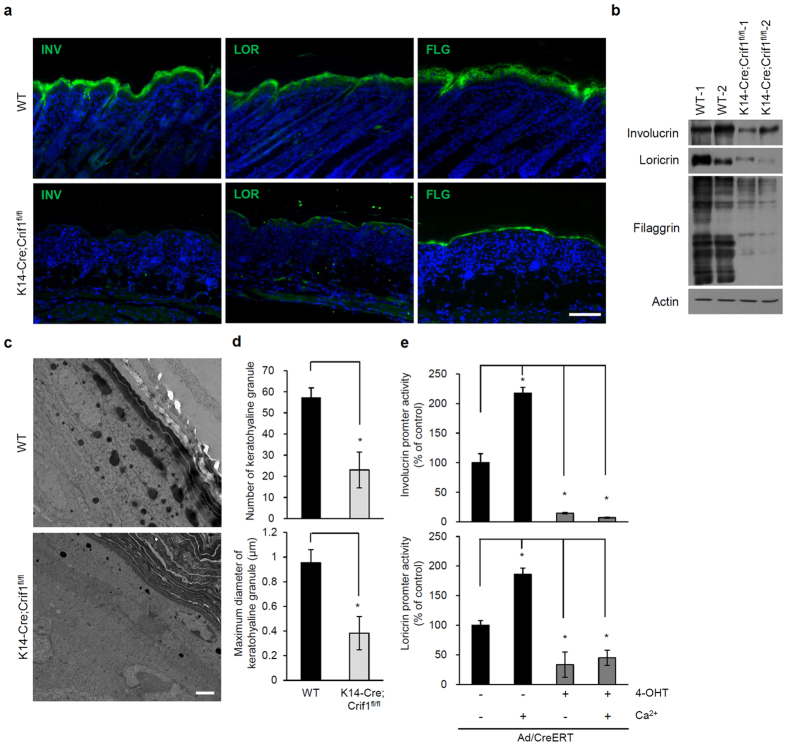
Impaired epidermal differentiation in *Crif1* cKO mice. (**a**) Immunofluorescence staining of epidermal differentiation markers, such as involucrin (INV), loricrin (LOR), and filaggrin (FLG) in skin sections of WT and *Crif1* cKO mice (P5) (green, IVN, LOR, and FLG; blue, DAPI). Scale bar, 100 μm. (**b**) Western blot analysis of epidermal lysates from WT and *Crif1* cKO mice (P5) to examine the levels of differentiation markers. Actin was used as a loading control. (**c**) Transmission electron microscopic images of the epidermis of WT and *Crif1* cKO mice (P5) showing keratohyaline granules in the granular layer. Scale bar, 2 μm. (**d**) Quantification of the number and maximum diameter of keratohyaline granules in WT and *Crif1* cKO. (**e**) Involucrin and loricrirn luciferase reporter activity in *in vitro Crif1* cKO model. To induce keratinocyte differentiation *in vitro*, cells were treated with 2 mM CaCl_2_ for 1 day (n = 3). *P < 0.01. Cropped blots were used in this figure and full-length blots are presented in Supplementary Figure 9.

As *Crif1*-deficient mice showed thinner epidermis with decreased granular layer, we further investigated the effect of CRIF1 levels on keratinocyte differentiation. First, we examined the expression of epidermal differentiation markers, such as involucrin, loricrin, filaggrin and K1, in the skin of WT and *Crif1* cKO mice. The epidermis of *Crif1* cKO mice showed markedly decreased expression of the differentiation markers, while that of WT mice displayed normal expression in the granular and cornified layers (Figs 3a, S5). Downregulation of differentiation markers was also confirmed by Western blot analysis (Fig. 3b). Keratohyalin is a protein found in the granular layer of the epidermis. In transmission electron microscopy, keratohyalin granules were frequently observed in the granular layer of WT mice, but the number and the maximum diameter of the keratohyalin granules were remarkably lower in *Crif1* cKO mice (Fig. 3c and d). These data show that *Crif1* is essential for keratinocyte differentiation.

To further study the roles of *Crif1*, we established an *in vitro Crif1* knockout model. Keratinocytes from *Crif1*^*fl/fl*^mice were cultured, transfected with an adenovirus expressing Cre-ERT, and treated with 4-hydroxy tamoxifen (4-OHT). *Crif1* was effectively knocked down in this model (Figure S6). Whether *Crif1* was required for calcium-induced keratinocyte differentiation was examined by involucrin and loricrin promoter assay. In *Crif1* cKO keratinocytes, involucrin and loricrin promoter activities were significantly decreased and were not induced by calcium treatment (Fig. 3e).

Keratinocyte differentiation is also important for embryonic development. To confirm whether *Crif1* is required for epidermal barrier function during embryonic development, we performed the skin permeability assay using toluidine blue staining at embryonic day 18.5. However, there was no difference in the skin permeability of WT and *Crif1* cKO embryos (Figure S7). These results suggest that *Crif1* was especially important for postnatal keratinocyte differentiation.

### Reduced epidermal proliferation and increased apoptosis in *Crif1* cKO

**Figure 4:**
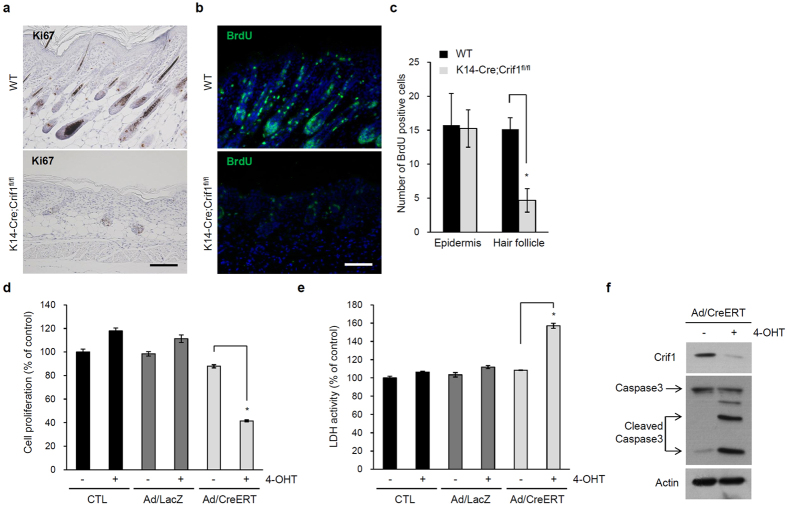
Reduced proliferation and increased apoptosis in *Crif1*cKO mice. (**a**) Immunohistochemistry of Ki67, a proliferation marker, in skin sections of WT and *Crif1* cKO mice (P5). (**b**) BrdU incorporation assay in WT and *Crif1* cKO mice (P5). Scale bar, 100 μm. (**c**) Quantification of the number of BrdU-positive cells in the epidermis and hair follicles of WT and *Crif1* cKO mice. (**d**) MTT assay in *Crif1* cKO *in vitro* model (n = 3). (**e**) LDH assay in *Crif1* cKO *in vitro* model (n = 3). *P < 0.01. (**f**) Western blot analysis for cleaved-caspase3 in *Crif1* cKO *in vitro* model. Actin was used as a loading control. Cropped blots were used in this figure and full-length blots are presented in Supplementary Figure 10.

As mitochondrial dysfunction is related to cell proliferation and apoptosis, we examined epidermal proliferation by immunohistochemical staining of Ki67 and BrdU incorporation assay. Ki67- and BrdU-positive cells were frequently detected in the basal layer of epidermis and hair follicles in WT mice, but fewer cells were detected in *Crif1* cKO mice (Fig. 4a and b). Interestingly, the number of BrdU-positive cells was particularly reduced in the hair follicles (Fig. 4c). Since hair follicles are highly proliferative during morphogenesis, mitochondrial function could be especially important in hair follicles. In addition, TUNEL-positive cells were observed in *Crif1*cKO mice, although they were rarely observed in WT mice (Figure S8). In agreement with the *in vivo* results, *Crif1* cKO keratinocytes exhibited impaired proliferation *in vitro* (Fig. 4d). To determine whether decreased proliferation was related to apoptosis, we performed the lactate dehydrogenase (LDH) assay which quantitatively measured LDH released into the medium from dead cells. LDH activity was significantly increased in *Crif1* cKO keratinocyte cultures (Fig. 4e). Similarly, cleaved caspase3, a marker of apoptotic cells, was also detected in *Crif1* cKO keratinocyte cultures (Fig. 4f).

### Down-regulation of Wnt/β-catenin signaling following loss of *Crif1*

**Figure 5:**
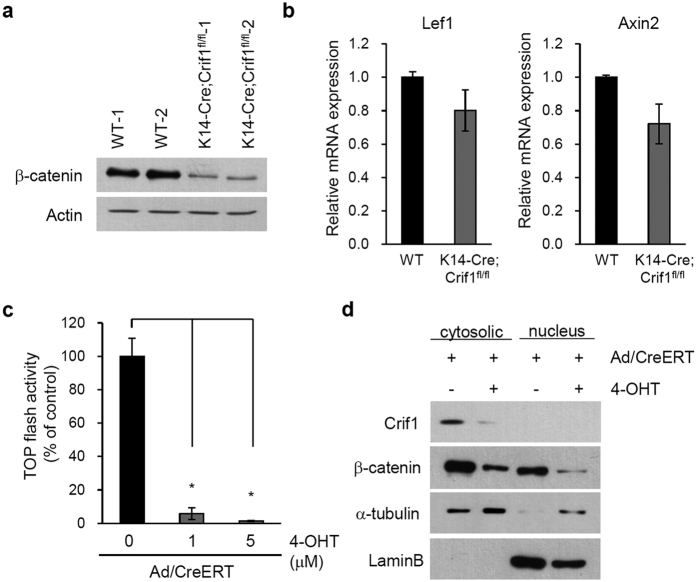
Down-regulation of Wnt/β-catenin signaling in *Crif1* cKO mice. (**a**) Western blot analysis for β-catenin using epidermal lysates from WT and *Crif1* cKO (P5). (**b**) qPCR analysis of Wnt/β-catenin downstream genes, *Lef1* and *Axin2*, using epidermal lysates from WT and *Crif1* cKO (P5). (**c**) TOP flash assay assessing transcriptional activity of β-catenin in *Crif1* cKO *in vitro*. *P < 0.01. (**d**) Cell extracts were fractionated and expression of β-catenin was assessed by Western blot. α-Tubulin and laminB were used for determination of the cytosolic and nuclear fractions, respectively. Cropped blots were used in this figure and full-length blots are presented in Supplementary Figure 11.

As Wnt/β-catenin signaling is essential for hair follicle morphogenesis and cycling *in vivo*^20^,^21^, we examined whether Wnt/β-catenin signaling was affected by loss of *Crif1*. Expression of β-catenin was significantly decreased in epidermal lysates from P5 *Crif1* cKO mice compared with that in cells from WT mice (Fig. 5a). We also observed reduced mRNA expression of β-catenin downstream genes, such as *Lef1* and *Axin2*, in the epidermis of *Crif1* cKO mice (Fig. 5b). Further, using TOPflash reporter assay, we investigated whether *Crif1* loss affected the transcriptional activation of β-catenin. TOPflash activity was significantly decreased following *Crif1* knockdown *in vitro* (Fig. 5c). And the protein level of both cytosolic and nuclear β-catenin was decreased by *Crif1* knockdown *in vitro* (Fig. 5d). These results suggest that defective hair morphogenesis was linked to down-regulation of Wnt/β-catenin signaling in *Crif1*cKO mice.

## Discussion

CRIF1 was first identified as a nuclear protein which regulates the function of several transcription factors. However, a recent study has revealed that CRIF1 is a mitochondrial protein, which is critical for mitochondrial function as it regulates the synthesis and insertion of OXPHOS polypeptides in the inner mitochondrial membrane. *Crif1*-null mouse embryonic fibroblasts (MEFs) show mitochondrial defect, based on the reduction in the levels of mtDNA-encoded proteins and the occurrence of morphological abnormalities such as loss of electron dense material from the matrix and distorted cristae^12^. CRIF1 contains several functional domains, including a mitochondrial targeting sequence, a nuclear localizing signal and coiled-coil domains. When CRIF1 is tagged with green fluorescent protein (GFP) at its C-terminal region, it is primarily localized in the mitochondria. In contrast, N-terminal GFP-tagged CRIF1 is only detected in the nuclei, suggesting that N-terminal tagging might prevent proper trafficking to the mitochondria. And mitochondrial dysfunction of *Crif1*-null MEFs is rescued by over-expressing the C-terminal tagged *Crif1* construct, but not by the N-terminal tagged *Crif1* construct. Therefore, it is suggested that mitochondrial function of CRIF1 is independent of its nuclear function^12^. Moreover, various tissues of *Crif1* cKO models display mitochondrial dysfunction. For example, cardiac muscle-specific *Crif1* cKO mice show cardiac hypertrophy, which is associated with mitochondrial damage in cardiomyocytes. Adipose tissue-specific *Crif1*-haploinsufficient mice exhibit reduced OXPHOS capacity and develop marked insulin resistance^12^,^13^. Therefore, targeted deletion of *Crif1* in specific tissues is a good approach to studying mitochondrial dysfunction.

It has been previously demonstrated that mitochondrial function is related to skin biology, such as skin aging, epidermal differentiation and hair development. Natural aging is accompanied by decreased mitochondrial activity and increased cellular senescence in human and mouse skin^22^,^23^,^24^. Moreover, mitochondrial oxidative stress promotes senescence of skin cells *in vivo*. Constitutive *Sod2* deficiency results in mitochondrial dysfunction and cellular senescence in the epidermis^25^. Epidermal differentiation and hair development are also regulated by mitochondrial reactive oxygen species (ROS). Deletion of *Tfam* in the epidermis results in reduced differentiation and progressive loss of hair follicles, as *Tfam*-deficient keratinocytes fail to generate mitochondrial ROS, which is required for Notch and β-catenin signaling^17^.

In this study, we generated epidermis-specific *Crif1* cKO mice to examine the effect of mitochondrial dysfunction in epidermis. The epidermis of *Crif1* cKO mice showed mitochondrial dysfunction, as observed by reduction in the expression of an mtDNA-encoded protein and abnormal mitochondrial morphology. This result demonstrates that *Crif1* is essential for regulating mitochondrial function in the epidermis.

Epidermal homeostasis is regulated by maintaining a balance between proliferation and differentiation of keratinocytes. *Crif1*-deficient keratinocytes exhibited impaired differentiation, in which calcium failed to activate the promoters of involucrin and loricrin. These results are consistent with the data obtained from another mitochondrial function- impaired mouse model, the *Tfam* cKO mouse. The *Tfam* cKO mice exhibit down-regulation of Notch signaling due to reduction in mitochondrial ROS^17^. Therefore, we hypothesized that the reduced differentiation seen in the epidermis of *Crif1* cKO mice could be related to Notch signaling.

In addition to impaired epidermal differentiation, *Crif1* cKO mice showed significantly decreased proliferation of hair follicles and increased apoptosis. Previous studies have shown that lack of *Crif1* is strongly associated with apoptosis. For example, *Crif1*^*-/-*^ embryos manifest developmental arrest, accompanied with defective proliferation and massive apoptosis, and die at around embryonic day 6.5^8^. Brain-specific *Crif1* cKO mice show neuronal degeneration and increased apoptosis^13^. Therefore, we conclude that *Crif1* deficiency-induced apoptosis of basal keratinocytes results in reduced terminal differentiation. This impaired epidermal homeostasis might cause defects in postnatal barrier function.

Previous studies have shown that inactivation of Wnt/β-catenin signaling causes hair growth defect. For example, deletion of β-catenin in the epidermis results in failure of anagen induction^21^,^26^; deletion of Wntless required for Wnt ligand secretion in epidermis causes delayed hair growth and regeneration^27^. Therefore, inhibition of Wnt/β-catenin signaling by *Crif1* deficiency could be a major reason for failure of hair follicle morphogenesis. Alternatively, mitochondrial dysfunction caused by *Crif1* loss can reduce the energy supply required for active hair growth.

In summary, we demonstrate the essential role of *Crif1* in hair morphogenesis and maintaining epidermal homeostasis. Mitochondrial dysfunction caused by loss of *Crif1* in the epidermis leads to impaired epidermal differentiation and defective hair morphogenesis. Our results contribute to the understanding of the importance of mitochondrial function in the regulation of epidermal homeostasis.

## Materials and Methods

### Mice

*Crif1*^*fl/fl*^C57BL/6 mice were generated as previously described^8^ and were crossed with *K14-Cre*B6CBAF1 mice purchased from the Jackson Laboratory (stock no. 004782). Heterozygous *K14-Cre;Crif1*^*fl*/+^mice were bred to *Crif1*^*fl/fl*^mice to obtain WT (*Crif1*^*fl/fl*^) and *Crif1* cKO (*K14-Cre;Crif1*^*fl/fl*^) mice. All experiments were performed in accordance with institutional guidelines, and were approved by the Institutional Review Board at Chungnam National University (CNU-00734).

### Primary keratinocyte culture and *in vitro* conditional knockout model

Primary keratinocytes were isolated and cultured from newborn mice as described^28^. Briefly, epidermis was separated from dermis by overnight incubation in trypsin without EDTA. The collected epidermis was minced and filtered through a cell strainer. Cells were cultured in Keratinocyte Growth Medium. pMSCV CreERT2 puro was purchased from Addgene (plasmid #22776) and was subjected to PCR cycles with primer set: forward (5′-AGCTGGTACCATGTCCAATTTACTGACCGT-3′) and reverse (5′-AGCTCTCGAGTCAGACTGTGGCAGGGAAAC-3′). A PCR product was subcloned into pENTR CMV vector that has attL sites for site-specific recombination with Gateway destination vector (Invitrogen). The replication-incompetent adenoviruses were created using Virapower adenovirus expression system (Invitrogen) according to the method previously described^29^. Briefly, site-specific recombination between entry vector and adenoviral destination vector was achieved by LR clonase (Invitirogen). The resulting adenoviral expression vector was then transfected into 293 A cells using Lipofectamine 2000 (Invitrogen). Cells were grown until 80% cytopathic effect (CPE) was seen, then harvested for preparation of recombinant adenovirus. For *in vitro* conditional knockout, cells were transduced with an adenovirus expressing Cre-ERT and treated with 4-hydroxy tamoxifen (4-OHT) (Sigma-Aldrich, H6278).

### Histology and immunofluorescence

Tissue samples were fixed with 10% formaldehyde, embedded in paraffin, and cut into 4-μm-thick sections. The sections were deparaffinized in xylene and then rehydrated using an alcohol series. IHC protein block (DAKO) and antibody diluents (DAKO) were used. The sections were then incubated overnight at 4 °C with primary antibodies. The following primary antibodies were used in this study: Involucrin (Abcam, ab28057, 1:200), Loricrin (Corvance, PRB-145P, 1:200), Filaggrin (Abcam, ab81468, 1:200), MTCO1 (Abcam, ab14705, 1:100), K1 (Santacruz, sc-65999, 1:100), and K14 (Santacruz, sc-53253, 1:100). The sections were incubated with fluorescence-conjugated secondary antibody at room temperature for 30 minutes. And they were finally visualized under a fluorescence microscope (Olympus Corporation, Tokyo, Japan).

### Oil Red O staining

Oil Red O staining solution was prepared by dilution of Oil Red O (0.3% in isopropanol) in dH2O (3:2) and filtered. To observe the subcutaneous fat and sebaceous glands using Oil Red O, frozen sections were washed in PBS, and rinsed in 60% isopropanol for 10 min. Sections were stained with Oil Red O solution for 5 min, rinsed with 60% isopropanol, counterstained with hematoxylin, and mounted with aqueous mounting medium.

### BrdU incorporation assay

To label the BrdU-positive cells *in vivo*, P5 mice were injected subcutaneously with a single dose of BrdU (Sigma-Aldrich, B5002, 50 μg/g). After 2 hours, dorsal epidermis was collected and fixed with 10% formaldehyde. Immunostaining was performed as above. Anti-BrdU antibody (Abcam, ab6326, 1:100) was used to determine proliferation index.

### Transmission Electron Microscopy

Tissue samples were fixed with 2.5% glutaraldehyde in PBS for 2 hours at 4 °C. After five washes with PBS, they were postfixed with 1% osmium tetroxide on ice for 2 hours and washed five times with PBS. And the tissues were embedded in EMbed-812 (EMS, USA) after dehydration in an ethanol and propylene oxide series. After polymerization of the resin at 70 °C for 2 days, serial sections were cut with a diamond knife on an ultramicrotome (UltraCut-UCT, Leica, Austria) and mounted on 100-mesh copper grids. Sections were stained with 2% uranyl acetate for 15 min and lead citrate for 5 min and examined under a transmission electron microscopy (TEM) (Technai G2 Spirit Twin, FEI, Hillsboro, USA) at 120 kV.

### Western blots

Dosal epidermal tissues were treated with dispase for overnight at 4 °C. The epidermis was separated and lysed in protein extraction solution (Intron) after homogenization using tissue lyser (QIAGEN). Epidermal extracts were centrifuged for 15 min at 15,000 rpm. Total protein was measured using a BCA protein assay kit (Thermo Scientific, Rockford, IL). Samples (20–30 μg protein per lane) were run on SDS-polyacrylamide gels, transferred onto nitrocellulose membranes and incubated with appropriate antibodies (1:1,000) for overnight at 4 °C with gentle agitation. Blots were then incubated with peroxidase-conjugated secondary antibodies (1:2,000) for 30 minutes at room temperature, and visualized by enhanced chemiluminescence (TransLab). The following primary antibodies were used in this study: Crif1 (Santacruz, sc-134882), β-catenin (Santacruz, sc-7199), Actin (Santacruz, sc-1615), LaminB (Santacruz, sc-6216), α-tubulin (Santacruz, sc-8035), MTCO1 (Abcam, ab14705), Involucrin (Abcam, ab28057), Filaggrin (Abcam, ab81468), and Loricrin (Corvance, PRB-145P).

### qRT-PCR

Total RNA was isolated from tissues using RNA mini kit (Ambion) and 4 μg was reverse-transcribed with moloney-murine leukaemia virus (M-MLV) reverse transcriptase (ELPIS biotech). qRT-PCR was performed on Applied Biosystems StepOne with SYBR Green real-time PCR master mix (Applied Biosystems) according to the manufacture’s protocol. Actin was used as an internal control. Each sample was examined in triplicate. The relative expression levels of mRNA were determined by normalizing with internal controls and calculated by using the comparative Ct method. The following primer sequences were used: *Crif1* (5′-GCGAAAGCAGAAGCGAGAAC-3′, 5′-GGCCCTCCGCTCCTTGT-3′), Axin2 (CCCTCCGGCAGCTATGAA, GGAGAGGTGGTCGTCCAAAA), Lef1 (5′-AGGGCGACTTAGCCGACAT-3′, 5′-TGCTGGCTGGGATGATTTC-3′), Actin (5′-CGATGCCCTGAGGCTCTTT-3′, 5′-TGGATGCCACAGGATTCCA-3′).

### Subcellular fractionation

Nuclear extracts from the cells were prepared using NE-PER Nuclear and Cytoplasmic Extraction Reagents (Thermo Scientific), according to the recommended protocol. To confirm the purity of subcellular fractionation, the extracts were Western blotted and probed with nuclear specific anti-laminB antibody.

### Luciferase assay

For production of involucrin-luc and loricrin-luc reporter adenoviruses, genomic DNA isolated from epidermal keratinocytes was used as a template for PCR. Primer sequences were as follows: involucrin promoter, 5′-CTCCATGTGTCATGGGATATG-3′ and 5′-TCAACCTGAAAGACAGAAGAG-3′; loricrin promoter, 5′ TACCAAGCAATCCTCTCACCTTGG-3′ and 5′-TGAGGAGAGAAGATGCTGGC-3′. The resultant PCR fragments cover from −2,467 to +1,239 base pairs of involucrin transcription site, and from −2,033 to +12 base pairs of loricrin transcription site. For production of TOPflash reporter adenovirus, the DNA fragment containing three copies of T-cell factor (TCF) binding site upstream of the thymidine kinase minimal promoter was amplified from TOPflash plasmid (kindly provided by Dr. Kwonseop Kim, Chonnam National University, Gwangju, Korea) with primers 5′-ATTCACGGTACCTATCATGTCTGGATCAGCC-3′ and 5′-ATTCACAAGCTTGGAGATCCTCTAGAGAGA-3′. The amplified fragment was subcloned into pGL3Promoter vector (Promega), then moved into pENT vector for Gateway cloning.

Primary keratinocytes were grown at 50% confluency in 12-well culture plate, then co-transduced with adenoviruses harboring CreERT and promoter reporters such as involucrin, loricrin and TOPflash. After incubation for 6 h, cells were replenished with fresh medium and treated with 4-OHT and/or calcium. Cells were further incubated for 1days. Luciferase activities were determined using Luciferase assay system (Promega), according to the recommended protocol.

### MTT assay and LDH assay

Cells were treated with 5 mg/ml MTT solution and were incubated for a further 30 min. The medium was removed and the resulting formazan crystal was solubilized in 250 μl of dimethylsulfoxide (DMSO). The optical density at 540 nm was determined using an ELISA reader. LDH activities were determined using Cytotoxicity detection kit (Roche), according to the recommended protocol.

### TUNEL assay

Paraffin-embedded tissue sections were used for TUNEL assay to determine the apoptotic cells in tissues. TUNEL-positive cells were detected using *In situ* Apoptosis Detection Kit (Abcam, ab206386), according to the recommended protocol.

### Statistical analysis

Data were evaluated statistically using one-way analysis of variance (ANOVA) using SPSS software (ver 22.0). Statistical significance was set at p < 0.01.

## Additional Information

**How to cite this article**: Shin, J.-M. *et al*. Targeted deletion of *Crif1* in mouse epidermis impairs skin homeostasis and hair morphogenesis. *Sci. Rep.* **7**, 44828; doi: 10.1038/srep44828 (2017).

**Publisher's note:** Springer Nature remains neutral with regard to jurisdictional claims in published maps and institutional affiliations.

## Supplementary Material

Supplementary Information
