# Supplementary material for: Targeted deletion of Crif1 in mouse epidermis impairs skin homeostasis and hair morphogenesis
Source: Sci Rep. 2017 Mar 20;7:44828. doi: 10.1038/srep44828 (PMC5357846; doi:10.1038/srep44828)
Supplement: Supplementary Information [file srep44828-s1.pdf]

Supplementary Information

Targeted deletion of *Crif1* in mouse epidermis impairs skin homeostasis and hair morphogenesis

Jung-Min Shin [1], Dae-Kyoung Choi [1], Kyung-Cheol Sohn [1], Ji-Young Kim [1], Myung Im [1], Young Lee [1], Young-Joon Seo [1], Minho Shong [2], Jeung-Hoon Lee [1], Chang Deok Kim [1]

[1] Department of Dermatology, Chungnam National University School of Medicine, Daejeon, Korea
[2] Division of Endocrinology, Department of Internal Medicine, Research Center for Endocrine and Metabolic Diseases, Chungnam National University School of Medicine, Daejeon, Korea

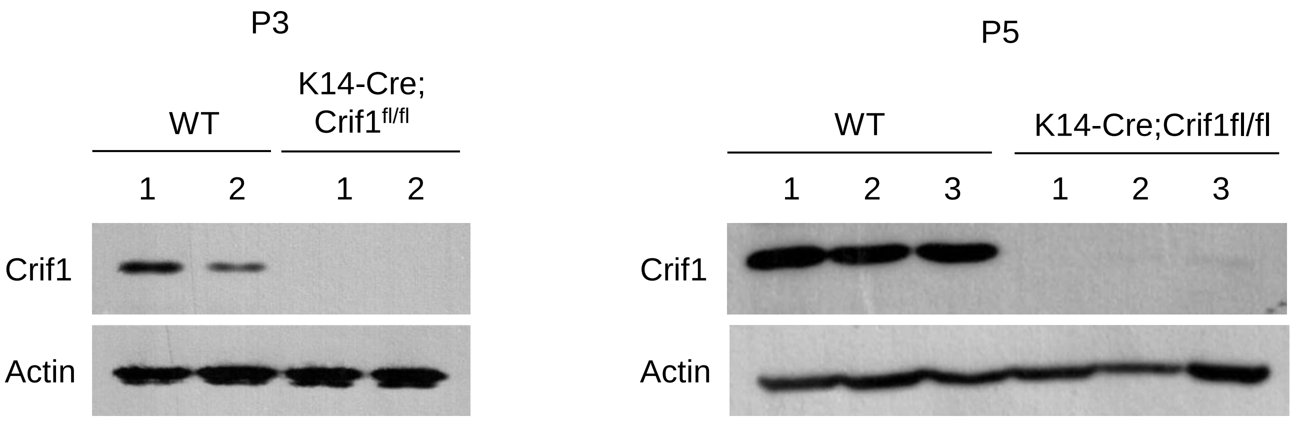

Supplementary Figure 1. Western blot analysis of Crif1 in epidermal lysates from WT and *Crif1* cKO mice at P3 and P5.

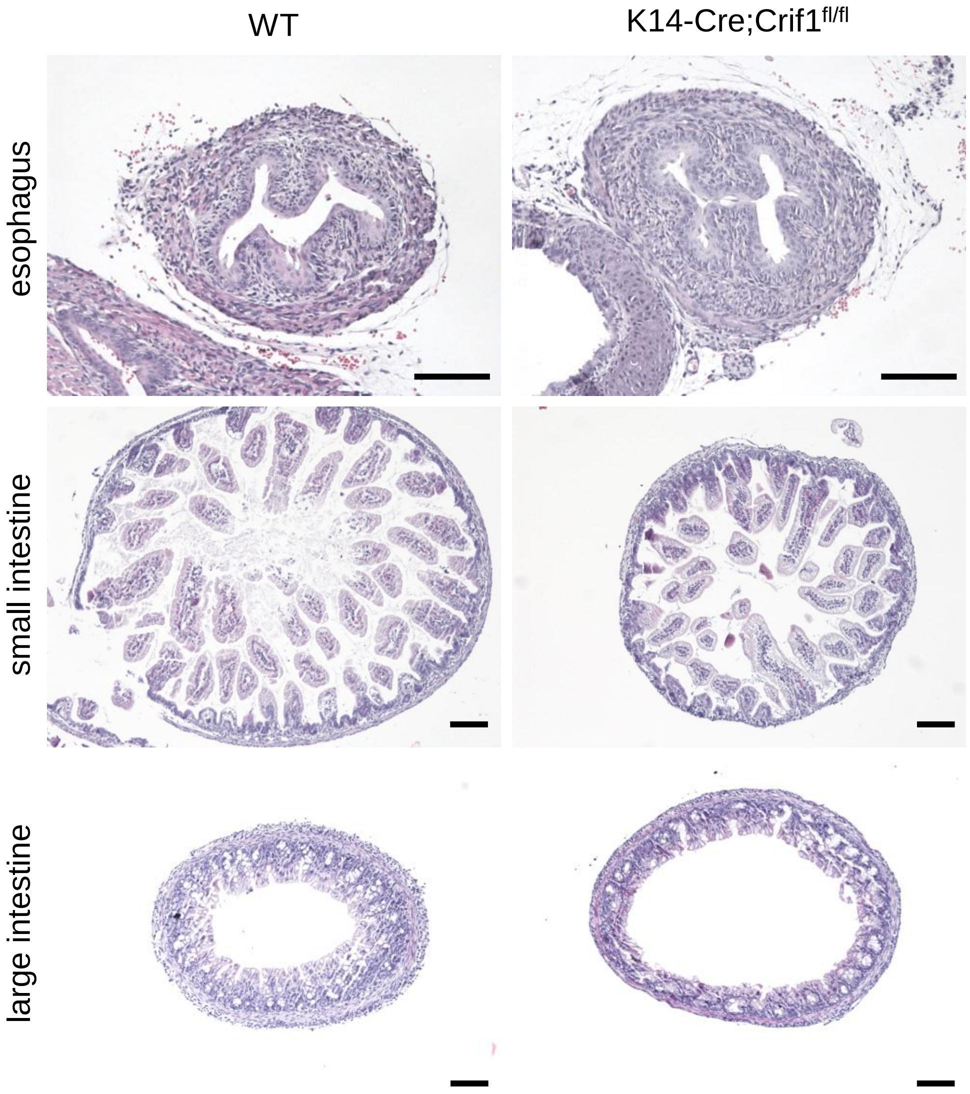

Supplementary Figure 2. Histological examination of epithelial tissues in *Crif1* cKO mice
Sections of epithelial tissues from WT and Crif1 cKO mice at P5 were stained with hematoxylin and eosin (H&E), observing no differences between WT and Crif1 cKO mice. Scale bar, 100 μm

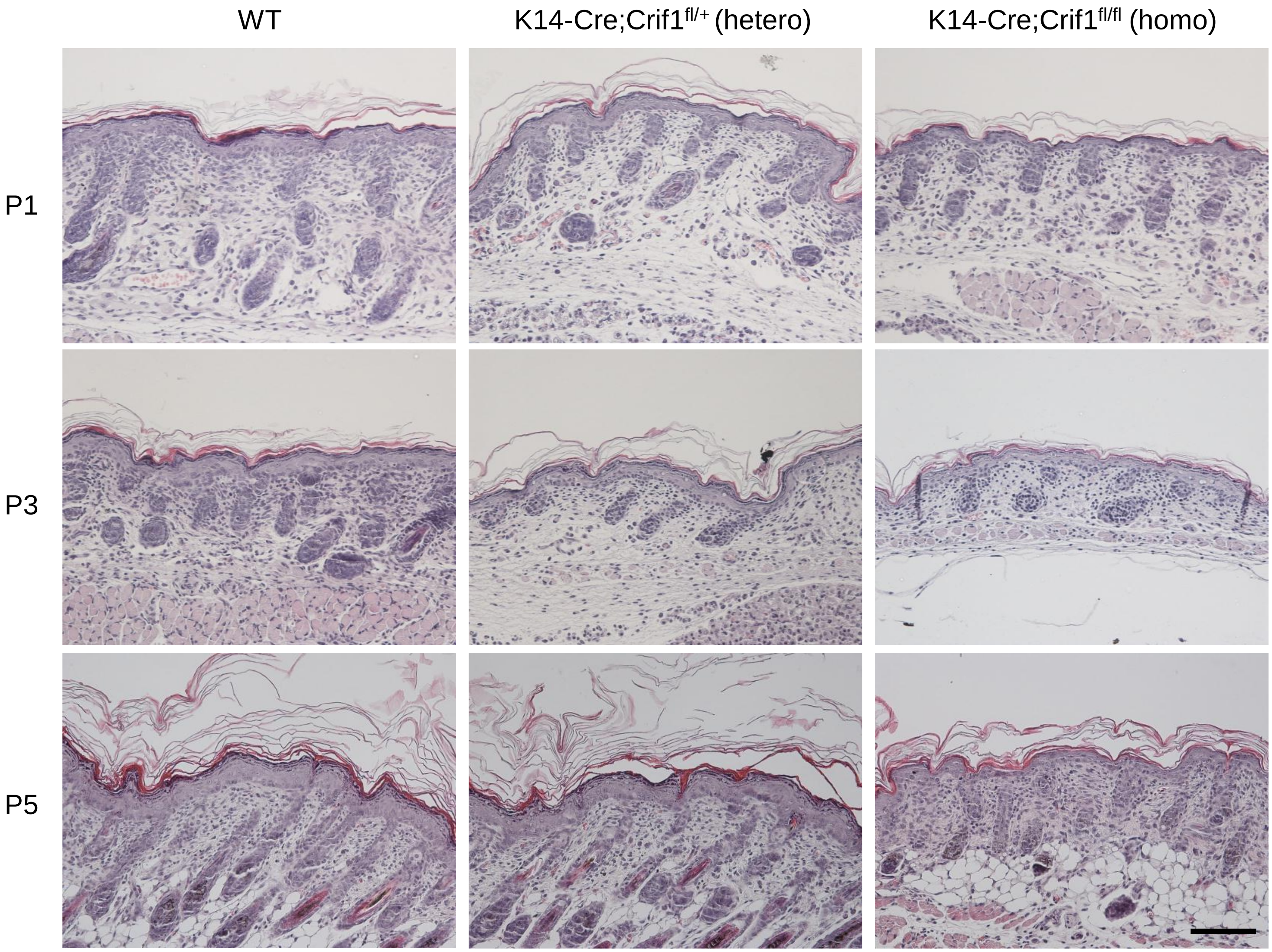

Supplementary Figure 3. Histological examination of WT, heteroxygous, and homo mice
There was no histological difference between heteroxygous (K14-Cre; Crif1$^{fl/+}$) and WT (Crif1$^{fl/fl}$) mice. Scale bar, 100 μm

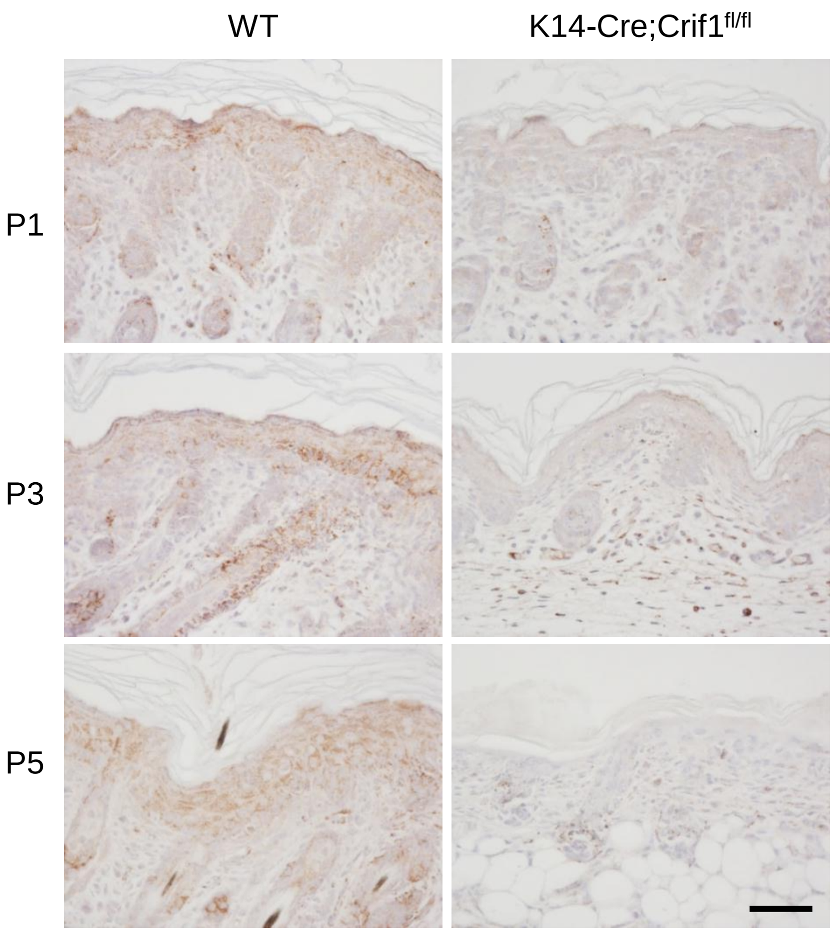

Supplementary Figure 4. Immunohistochemical staining of mt-Co1 in epidermis of WT and *Crif1* cKO mice.
Scale bar, 200 μm.

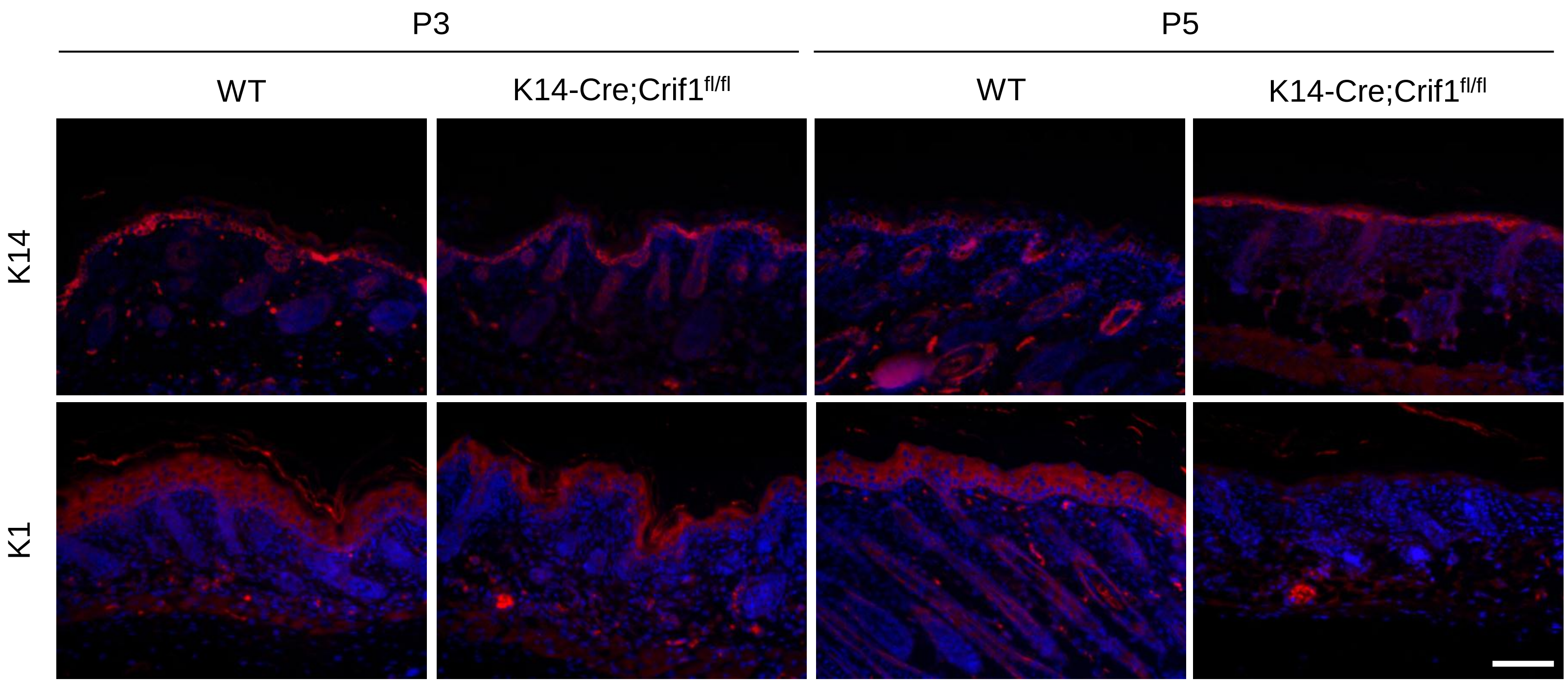

Supplementary Figure 5. Immunofluorescence staining of K14 and K1 in epidermis of WT and *Crif1* cKO mice.
Scale bar, 100 μm

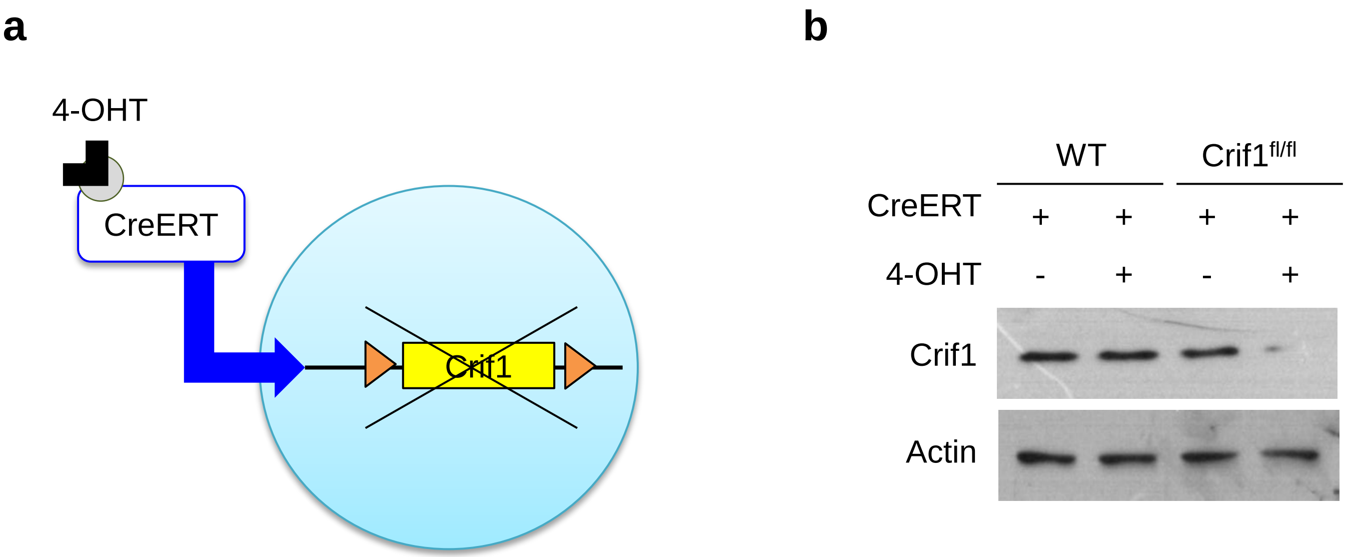

Supplementary Figure 6. Establishment of *Crif1* knockout model in vitro

Primary keratinocytes cultured from Crif1$^{fl/fl}$ mice were transduced with an adenovirus expressing CreERT (5 MOI) for 6 hrs. Cells were replenished, treated with 4-OHT and then cultured for a further 2 days. The expression of Crif1 was effectively reduced by 4-OHT treatment only in Crif1$^{fl/fl}$ cells.

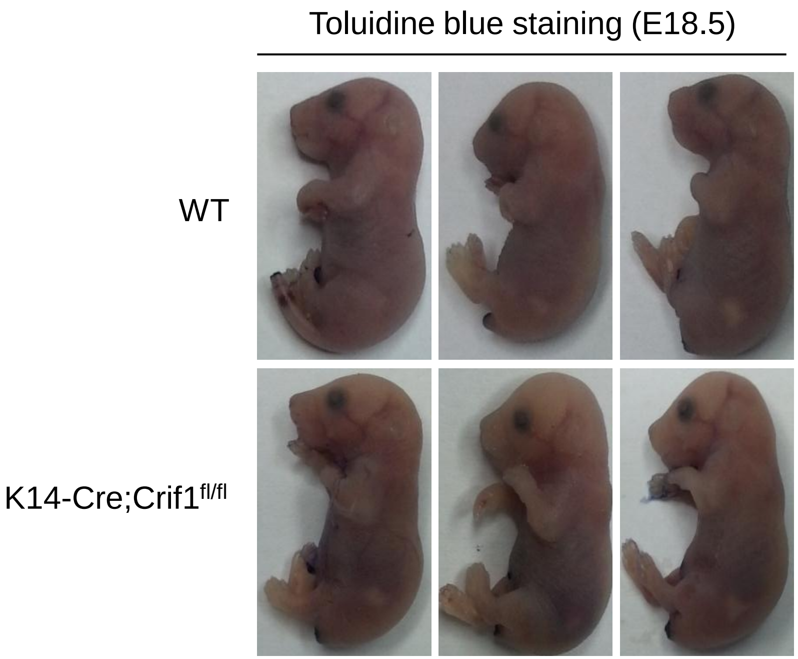

Supplementary Figure 7. Effect of Crif1 on skin barrier in embryonic development
Toluidine blue staining was performed to examine the barrier defect in embryonic development. (embryonic day18.5) Crif1 cKO embryos showed normally developed epidermis.

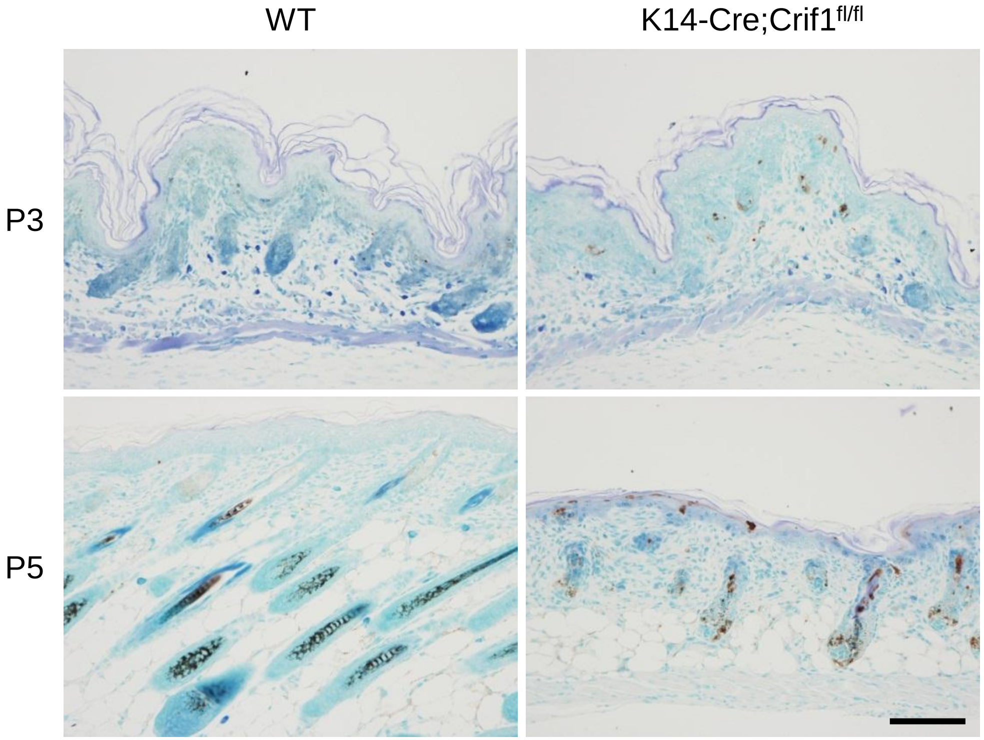

Supplementary Figure 8. TUNEL assay in epidermis of WT and *Crif1* cKO mice.
To examine the apoptotic cells, TUNEL assay was performed in epidermis of WT and Crif1 cKO mice at P3 and P5.
Scale bar, 100 μm

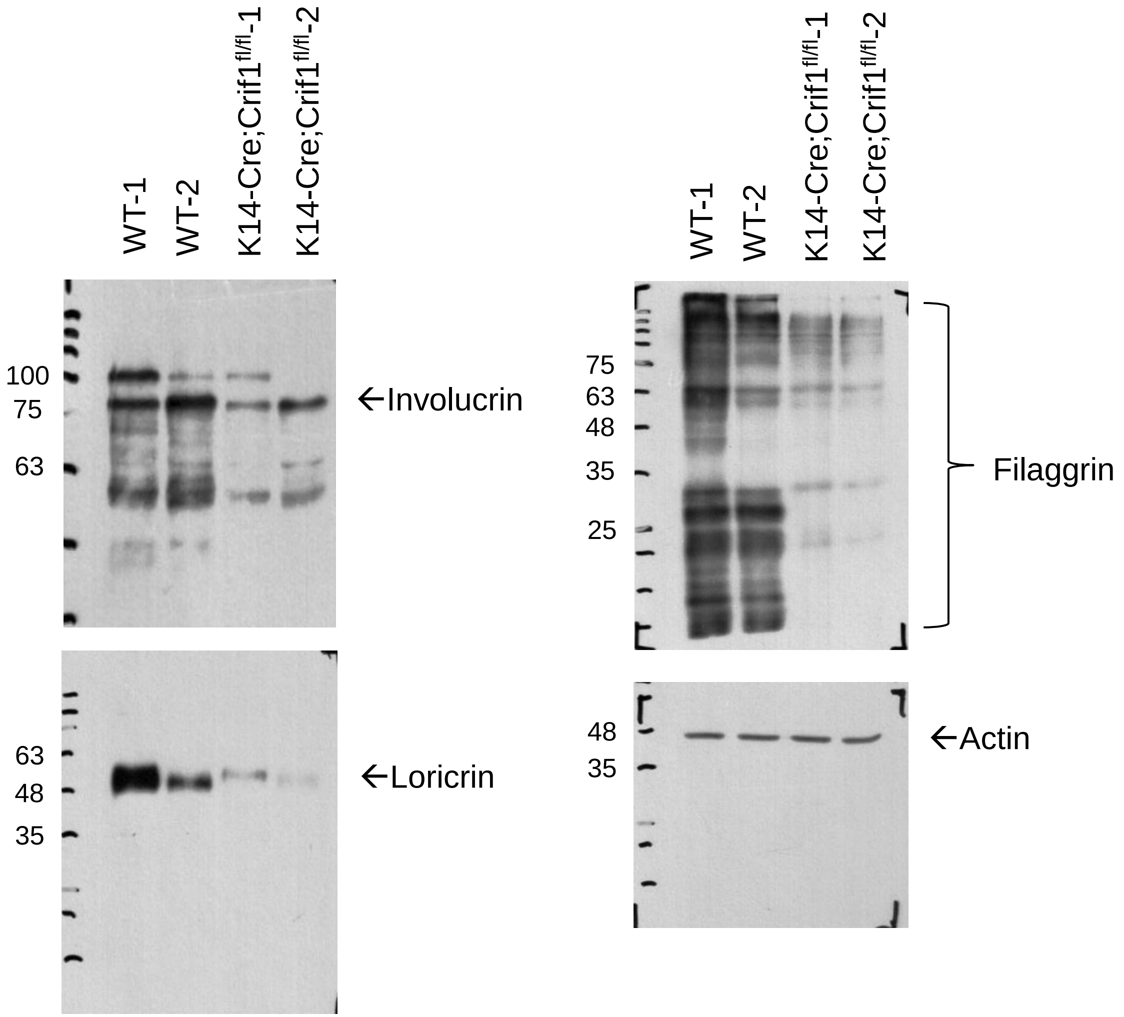

Supplementary Figure 9. Uncropped data for Figure 3b

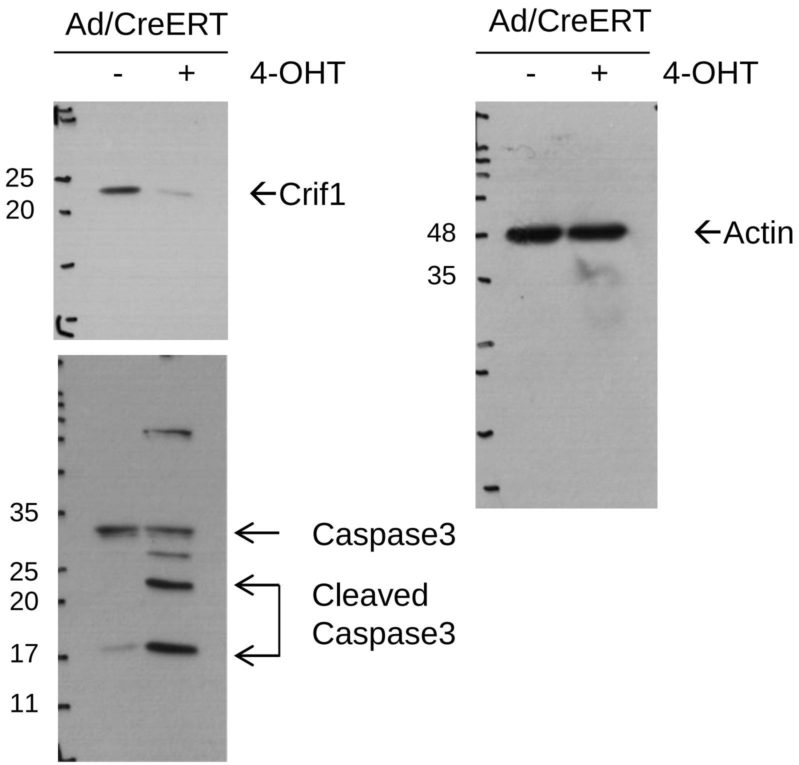

Supplementary Figure 10. Uncropped data for Figure 4f

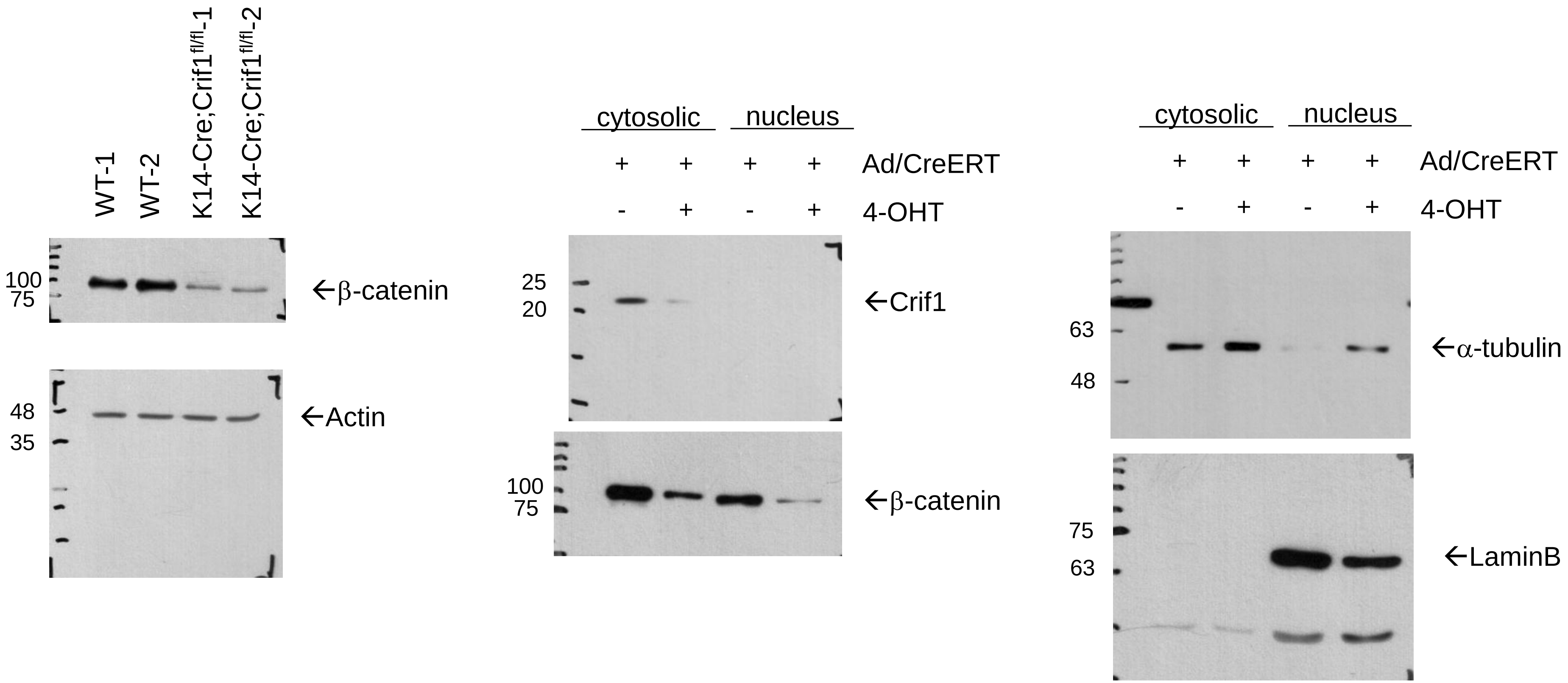

Supplementary Figure 11. Uncropped data for Figure 5a and d
